# Desensitization treatment in MIH-affected teeth: a systematic review

**DOI:** 10.1007/s40368-024-00934-2

**Published:** 2024-08-13

**Authors:** Ebba Hjertberg, Adnan Hajdarević, Birgitta Jälevik

**Affiliations:** 1 Institute of Odontology, Sahlgrenska Academy, University of Gothenburg, Gothenburg, Sweden; 2 Department of Pediatric Dentistry, Institute of Odontology at the Sahlgrenska Academy, University of Gothenburg, Gothenburg, Sweden; 3 Folktandvården Björkekärr, Public Dental Service, Region Västra Götaland, Gothenburg, Sweden

**Keywords:** Dental enamel defect, Pain, Tooth sensitivity, Pediatric dentistry, Treatment modalities

## Abstract

**Purpose:**

The present systematic review aims to summarize the current evidence regarding various treatment modalities and their results when dealing with hypersensitivity in teeth with Molar-Incisor Hypomineralization (MIH).

**Methods:**

Systematic searches were conducted in PubMed and Scopus using the search terms "MIH AND Hypersensitivity AND treatment." Studies involving children aged 6–18 years diagnosed with MIH and exhibiting hypersensitivity were considered for inclusion. The outcomes examined included clinical, behavioral, and psychosocial treatment options for reducing hypersensitivity. A meta-analysis was performed for six of the included articles, and the *I*^2^ value was calculated to determine heterogeneity.

**Results:**

A total of eight articles met the inclusion criteria for this review, with six eligible for the meta-analysis. Various treatment modalities, such as dental mousse, sealing, laser therapy, and crown therapy, demonstrated significant reductions in hypersensitivity individually (*p* < 0.05).

**Conclusion:**

The treatment methods that offer the most effective reduction in hypersensitivity are sealing with resin composite or glass ionomer cement for mild cases of MIH, while crown therapy is more effective for severe cases of MIH. However, the small number of participants and short follow-up times limit the strength of these conclusions.

## Introduction

In the 1980s, observations of hypomineralized enamel areas in first permanent molars (FPM) at eruption were primarily documented (Koch et al. 1987; Suckling et al. 1987). Over the past two decades, this condition, termed Molar-Incisor Hypomineralisation (MIH) (Weerheijm et al. 2001), has garnered increasing attention worldwide. While primary molars and permanent incisors can exhibit similar opacities, they are not considered prerequisites for or involved in the diagnosis of MIH (FDI Working Group 1992). Despite numerous studies on the etiology of MIH, it remains obscure, with speculation that genetic and systemic factors may interact (Lygidakis et al. 2022; Garot et al. 2022).

The condition MIH is due to a disruption in enamel mineralization and primarily affects 1–4 of the first permanent molars, with potential extension to the permanent incisors (Weerheijm et al. 2001). Clinically, porous hypomineralized areas manifest as well-defined opaque spots ranging from white yellow to brownish. Enamel may disintegrate in areas with inadequate mineralization, especially at the occlusal parts of the teeth (Weerheijm et al. 2003). Children with MIH often experience pain and hypersensitivity, particularly during eating, tooth brushing, and inhalation of cold air, leading to a compromised oral health-related quality of life (Jälevik et al. 2022). The reason for hypersensitivity in teeth affected by MIH is still not fully understood (Fagrell et al. 2011). Rodd et al. (2007) hypothesized that the high porosity of hypomineralized enamel facilitates bacterial penetration into the dentinal tubules, leading to subclinical pulpal inflammation. Additionally, there is a scarcity of clinical studies conducted in a standardized manner that address the prevalence or intensity of dental hypersensitivity in MIH-affected teeth (Raposo et al. 2019).

In a recent study, Linner et al. (2021) concluded that tooth hypersensitivity is a significant clinical issue in children affected by MIH, with a higher degree of hypersensitivity observed in individuals aged 8 years and older, with molar teeth, and teeth affected by hard tissue breakdowns. Restoration of disintegrated teeth has been challenging due to insufficient anesthesia and failed fillings (Jälevik and Klingberg 2002; William et al. 2006). The prevalence of hypersensitivity in children with MIH has been reported to be around 35% (Raposo et al. 2019). Recently, the management of hypersensitivity in MIH teeth has garnered increased attention, leading to the publication of several studies. However, there are no reviews regarding this subject.

Systematic reviews are crucial for accurately and reliably summarizing evidence. This review aims to consolidate the current evidence regarding various methods for managing hypersensitivity in Molar-Incisor Hypomineralisation (MIH)-affected teeth in children and adolescents. Through a systematic approach, we seek to provide a comprehensive overview of the available literature, aiding clinicians, and researchers in making informed decisions regarding the management of this condition.

## Method

This systematic review adhered to a protocol in alignment with the Preferred Reporting Items for Systematic Reviews and Meta-Analysis (PRISMA) guidelines. The review protocol was registered in PROSPERO under the registration number 517264. The primary research question addressed in this review is: What treatment modalities are utilized to reduce hypersensitivity in teeth affected by Molar-Incisor Hypomineralisation (MIH), and what is the effectiveness of these therapies?

### Searches

Searches were conducted in both MEDLINE/PubMed and Scopus databases. A comprehensive search strategy was implemented, combining MeSH terms, MeSH synonyms, and free terms. The Boolean operators 'AND' and 'OR' were utilized to combine keywords effectively (Tables 1, 2). Searches were conducted from the inception dates of the databases to the date specified. Following selection, the references were scrutinized based on the predetermined eligibility criteria. The search was limited to peer-reviewed articles and not included gray literature.

**Table 1:** Search strategy for PubMed (2022-12-10)

Search order	Search string	No result
#5	(((MIH OR Molar Incisor Hypomineralization) AND (Hypersensitivity OR hypoplasia OR sensitivity)) AND (enamel OR teeth OR tooth OR molar)) AND (treatment OR therapeutics OR therapy OR silver diamine fluoride OR silver modifies atraumatic treatment OR restorative treatment OR casein phosphopeptide amorphous calcium phosphate OR laser theray)	221
#4	treatment OR therapeutics OR therapy OR silver diamine fluoride OR silver modifies atraumatic treatment OR restorative treatment OR casein phosphopeptide amorphous calcium phosphate OR laser theray	13,401,190
#3	enamel OR teeth OR tooth OR molar	345,615
#2	Hypersensitivity OR hypoplasia OR sensitivity	3,155,634
#1	MIH OR Molar Incisor Hypomineralization	1393

**Table 2:** Search strategy for Scopus (2022-12-10)

Search order	Search string	No result
#5	(((MIH OR Molar Incisor Hypomineralization) AND (Hypersensitivity OR hypoplasia OR sensitivity)) AND (enamel OR teeth OR tooth OR molar)) AND (treatment OR therapeutics OR therapy OR silver diamine fluoride OR silver modifies atraumatic treatment OR restorative treatment OR casein phosphopeptide amorphous calcium phosphate OR laser therapy)	172
#4	treatment OR therapeutics OR therapy OR silver diamine fluoride OR silver modifies atraumatic treatment OR restorative treatment OR casein phosphopeptide amorphous calcium phosphate OR laser therapy	5,263,315
#3	enamel OR teeth OR tooth OR molar	747,419
#2	Hypersensitivity OR hypoplasia OR sensitivity	2,815,313
#1	MIH OR Molar Incisor Hypomineralization	717

### Selection process

**Fig. 1:**
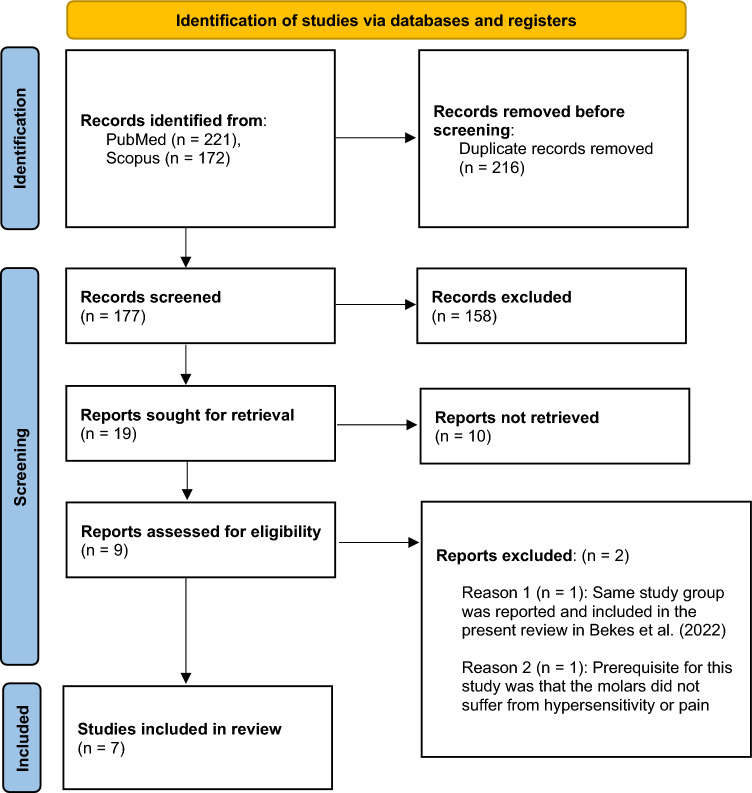
PRISMA flow diagram modified according to Page et al. (2021)

Duplicates were removed, and abstracts were screened to identify potential articles for inclusion. Subsequently, the authors (B.J. and E.H.) thoroughly reviewed the full text of the selected articles. Following comprehensive reading and discussion, any articles that did not meet the inclusion criteria were excluded (Fig. 1).

#### Inclusion criteria

- Full-text articles published in peer-reviewed journals.
- Diagnosis of Molar-Incisor Hypomineralisation (MIH) according to established criteria, such as the DDE index (FDI Working Group 1992) or EAPD index (Weerheijm et al. 2003).
- Articles written in the English language.
- Children aged 6–18 years diagnosed with MIH and exhibiting hypersensitivity.
- The PICO(T) Strategy Applied for Inclusion Criteria:
  o P—Population: Children diagnosed with Molar-Incisor Hypomineralization.
  o I—Intervention: Treatment modalities aimed at reducing hypersensitivity in MIH-affected teeth.
  o C—Comparison: Comparison of different treatment modalities or control groups.
  o O—Outcome: Reduction in hypersensitivity in MIH-affected teeth.

#### Exclusion criteria

- MIH diagnosis not in accordance with established MIH criteria.
- Studies focusing solely on incisors.
- Systematic reviews, letters to the editor, and case reports.
- Studies not using the Schiff Cold Air Sensitivity Scale (SCASS) for hypersensitivity measurement.

### Assessment of risk of *bias* in the included studies

The two authors (B.J. and E.H.) independently evaluated the eligible articles in full length, following comprehensive reading and discussion until full agreement was established. This assessment was conducted using Cochrane Risk of Bias Tool (Higgins et al. 2011) for randomized trials, and Cochrane Risk Of Bias In Non-Randomized Studies of Interventions (Sterne et al. 2016) for non-randomized studies.

### Data collection and data analysis

#### Population

- The country where the study was conducted.
- Type of study.
- Size and age of the study group.
- MIH diagnosis and hypersensitivity.
- The number of teeth with MIH diagnosis.
- The number of teeth with hypersensitivity.

#### MIH diagnosis

- Method for measuring pain and hypersensitivity.
- Type of treatment.
- MIH severity.

### Statistical analysis

A meta-analysis was performed utilizing Forest Plot to compare the difference in hypersensitivity reduction before and after treatment across various articles and their corresponding control groups in IBM SPSS Statistics version 27.0 (Statistical Package for the Social Sciences; SPSS, Chicago, IL, USA). The I^2^ statistic was calculated to assess heterogeneity.

## Results

### Data searches

After performing the database searches and excluding duplicates, 216 articles remained. Following abstract screening, 9 studies met the inclusion criteria, while 10 articles were excluded. The most common reason for exclusion was the absence of reporting hypersensitivity or pain as outcomes of treatment efficacy (Biondi et al. 2017; Durmus et al. 2021; Gatón-Hernandéz et al. 2020; Nogueira et al. 2021; Özgül et al. 2022). Additionally, two studies were excluded, because they did not meet the inclusion criteria for study type (Bakkal et al. 2017; da Silva et al. 2022). Furthermore, two studies were excluded as they only examined incisors (Özgül et al. 2013; Sezer and Kargul 2022). After full-text screening, two additional studies were excluded. Tugcu et al. (2022) was excluded, since a prerequisite for this study was that the molars did not suffer from hypersensitivity or pain. Additionally, Bekes et al. (2021) were excluded, because the same study group was reported and included in the present review in Bekes et al. (2022). One study was excluded for not using SCASS for measuring hypersensitivity (Muniz et al. 2020). As a result, 7 studies remained included in the present review (Fig. 1; Tables 3, 4).

**Table 3:** Characteristics of the studies included in the systematic review

Author and year	Country	No. of participants	Age of participants	Inclusion criteria	No. of teeth assessed	Study design	Sensitivity scale
Ballikaya et al. (2022)	Turkey	45	6–13 yrs	At least two hypersensitive MIH-affected first permanent molars were defined by an air-blast test, resulting in a SCASS score of two or three	56 in each study group	RCT, Split mouth, Consort	SCASS
Bekes et al. (2022)	Austria & Germany	38	6–10 yrs	The presence of demarcated opacities, post-eruptive enamel breakdown, or atypical restorations due to MIH in at least one first permanent molar	38 in each study group	Split mouth	SCASS
Bekes et al. (2017)	Austria & Germany	19	6–14 yrs	At least one hypersensitive molar with MIH, which had a qualifying response to air-blast stimuli applied for 1 s, as defined by a score of 2 or 3 on the SCASS	56	Non-randomized, single group study	SCASS, WBFS
Ehlers et al. (2021)	Germany	21	6–16 yrs	The presence of at least one hypersensitive MIH-affected molar that responds to a tactile stimulus with a score greater than 0 on the WBFS and to an air-blast stimulus with a score of 2 or 3 on the SCASS	48 study group 25 controls	Randomized, double-blind, case–control clinical trial	SCASS, WBFS, VAS
Pasini et al. (2018)	Italy	40	8–13 yrs	Teeth affected by MIH that had a SCASS score of ≤ 3 were selected for the study	40	Case–control	SCASS VAS
Rolim et al. (2021)	Brazil	35	7–16 yrs	Teeth requiring restorative treatment due to MIH with post-eruptive breakdown, previous unsuccessful atypical restorations, with or without carious lesions	64	RCT	Scass, Venham picture test, Faces pain scale- revised
Singh et al. (2022)	India	60	8–15 yrs	FPMs with hypomineralization involving a minimum of three cusps or the entire occlusal surface were selected	Not specified	Randomly distributed 3 groups. Lithium disilicate, Zirconia and full cast metal crowns. Consort	SCASS, VAS

**Table 4:** Excluded studies, and reasons

Author and year	Motivation
Bakkal et al. (2017)	Pilot study
Bekes et al. (2021)	Hypersensitivity and pain not measured
Biondi et al. (2017)	Hypersensitivity and pain not measured
da Silva et al. (2022)	Case report
Durmus et al. (2021)	Hypersensitivity and pain not measured
Gatón-Hernandéz et al. (2020)	Hypersensitivity and pain not measured
Nogueira et al. (2021)	Hypersensitivity and pain not measured
Muniz et al. (2020)	Not using SCASS for measuring hypersensitivity
Sezer and Kargul (2022)	Only incisors included
Tugcu et al. (2022)	Hypersensitivity and pain excluded
Özgül et al. (2013)	Only incisors included
Özgül et al. (2022)	Hypersensitivity and pain are excluded

### Risk of *bias*

**Fig. 2:**
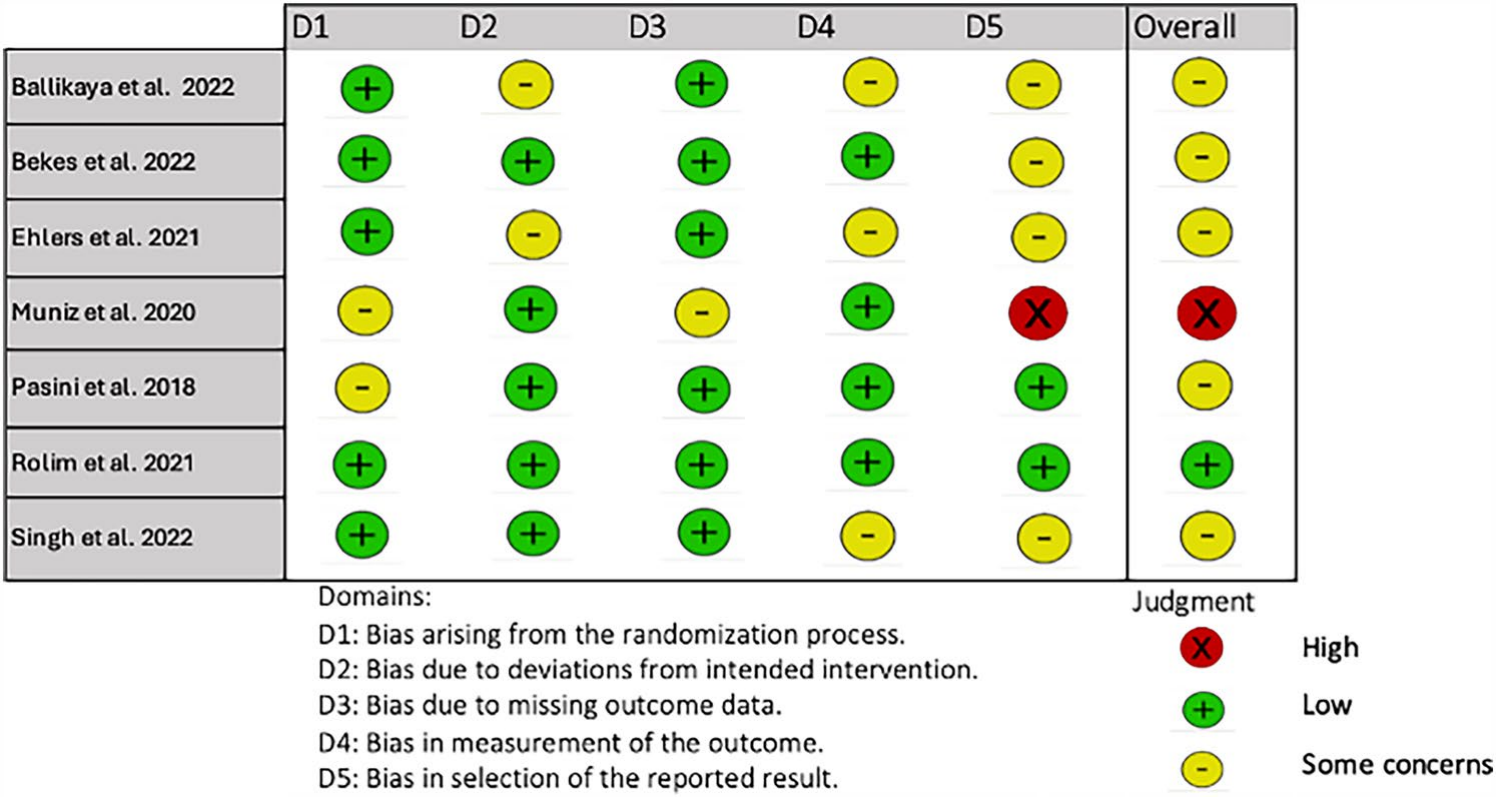
Risk-of-bias assessment for randomized clinical trials

For the randomized studies, the risk of bias was assessed using the Cochrane Risk of Bias Tool (Higgins et al. 2011). The different domains are detailed in Fig. 2. Among the seven randomized-controlled trials, one was assessed as low risk, five had some concerns, and one was at high risk of bias. The study by Muniz et al. (2020) was considered to have a high risk of bias due to issues in the selection of the reported result. Two studies lacked proper randomization processes and were thus assessed with a moderate risk of bias (Muniz et al. 2020; Pasini et al. 2018). A significant concern was bias in the selection of the reported result, with only Pasini et al. (2018) being assessed as low risk. The main issue was the inadequate reporting of results, as many studies only presented mean values. Consequently, Muniz et al. (2020) were excluded from this review due to high risk of bias and the absence of SCASS usage for measuring hypersensitivity (Fig. 2).

**Fig. 3:**
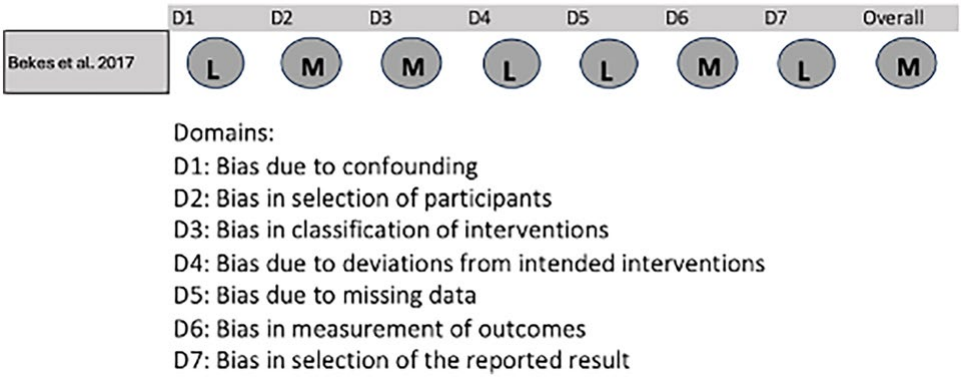
Risk-of-bias assessment for non-randomized trails

Bekes et al. (2017) did not include a control group, so the risk of bias was assessed using the Cochrane Risk Of Bias In Non-Randomized Studies of Interventions tool (Sterne et al. 2016) (Fig. 3). This study exhibited deficiencies in the selection of participants, classification of interventions, and measurement of outcomes, resulting in an overall moderate risk of bias. However, Bekes et al. (2017) were assessed to be at low risk of bias in the four other domains (Fig. 3).

### Populations

The size of the study groups and the age range of children varied across the included studies. The total age range observed was from 6 to 18 years. The size of the study groups ranged from 19 to 66 participants. Additionally, the number of treated teeth varied considerably among the studies (Table 5).

**Table 5:** Summary of the results of the included studies

Author and year	Method	Study group	Method specification	Hypersensitivity before treatment: mean (SD)	Hypersensitivity after treatment: mean (SD)	p value: total hypersensitivity reduction	P value: difference in hypersensitivity reduction: control and study group
Ballikaya et al. (2022)	Sealents	Study Gr. 1	Silver Diamine Fluoride (SDF)	SCASS: 1.77 (0.83)	SCASS: 0.15 (0.38)	*p* < 0.001	*p* > 0.05
		Study Gr. 2	Silver-Modified Atraumatic Restorative Treatment (SMART)	SCASS: 1.77 (0.83)	SCASS: 0.08 (0.28)	*p* < 0.001	
Bekes et al. (2022)	Sealents	Study Gr. 1	Clinpro Sealant in combination with Scotchbond Universal	SCASS: 2.3 (0.5) VAS: 7.1 (1.7)	SCASS: 0.1 (0.4) VAS: 0.8 (1.4)	*p* < 0.001	*p* > 0.05
		Study Gr. 2	Ketac Universal	SCASS: 2.4 (0.5) VAS: 7.1 (2.0)	SCASS: 0.1 (0.5) VAS: 0.8 (1.3)	*p* < 0.001	
Bekes et al. (2017)	Desensitizing pastes	Self-controls	Elmex Sensitive Professional toothpaste (8% arginine, calcium carbonate with 1450 ppm fluoride)	SCASS: 2.1 (0.3) WBFS: 2.1 (2.6)	SCASS: 0.8 (0.9) WBFS: 0.6 (1.1)	*p* < 0.001	N/A
Ehlers et al. (2021)	Desensitizing pastes	Study Gr	Intervention toothpaste with 10% microcrystalline hydroxyapatite was a commercially available product (Kinder Karex Zahnpasta)	WBFS: 5.6 (-) SCASS: 2.2 (-)	WBFS: 2.6 (-) SCASS: 1.0 (-)	Missing data	p = 0.75
		Ctr Gr	Control toothpaste with amine fluoride (1400 ppm F) (Elmex Junior Zahnpasta)	WBFS: 5.1 (-) SCASS: 2.0 (-)	WBFS: 3.4 (-) SCASS: 0.96 (-)	Missing data	
Pasini et al. (2018)	Desensitizing pastes	Study Gr	Recaldent CPP-ACP Tooth Mousse (casein phosphopeptide-amorphous calcium phosphate)	SCASS: 2.4 (0.6) VAS: 7.8 (1.0)	SCASS: 1.1 (0.4) VAS: 3.8 (0.6)	*p* < 0.001	*p* < 0.001
		Ctr Gr	Fluoride toothpaste	SCASS: 2.3 (0.5) VAS: 7.5 (1.3)	SCASS: 2.2 (0.4) VAS: 7.2 (0.8)	p = 0.49	
Rolim et al. (2021)	Restorations	Study Gr. 1	Total-etch (TE—37% phosphoric acid etching)	FPS-R, air blast: 2.12 (1.2)	FPS-R, air blast: 0.31 (0.54)	*p* < 0.05	*p* > 0.05
		Study Gr. 2	self-etch (SE—no prior etching)	FPS-R, air blast: 2.71 (2.4)	FPS-R, air blast: 0.39 (0.65)	*p* < 0.05	
Singh et al. (2022)	Full coverage crowns	Study Gr. 1	Metal crowns	SCASS: 2.25 (-)	SCASS: 0.0 (-)	*p* < 0.001	*p* > 0.05
		Study Gr. 2	Zirconia	SCASS: 1.4 (-)	SCASS: 0.0 (-)	*p* < 0.001	
		Study Gr. 3	Lithium disilicate	SCASS: 2.2(-)	SCASS: 0.0 (-)	*p* < 0.001	

### Diagnosing MIH

All studies, except for Pasini et al. (2018) utilized the EAPD index for diagnosing MIH (Weerheijm et al. 2003). Pasini et al. (2018) employed the modified DDE index (FDI Working Group 1992).

### Measuring hypersensitivity

Out of the eight reviewed studies, seven utilized the Schiff Cold Air Sensitivity Scale (SCASS) as the measurement method for teeth hypersensitivity. Among these, five studies complemented SCASS measurements with additional pain assessment tools, such as the Visual Analog Scale (VAS), Wong-Baker Faces Scale (WBFS), or Venham scale.

#### SCASS

The Schiff Cold Air Sensitivity Scale (SCASS) is a tool used to measure teeth hypersensitivity, specifically in response to air-blast stimuli. This scale assesses subject response to the stimulus and is scored as follows:

0: Subject does not respond to the air stimulus. 1: Subject responds to the air stimulus but does not request discontinuation of the stimulus. 2: Subject responds to the air stimulus and requests discontinuation or moves away from the stimulus. 3: Subject responds to the air stimulus, perceives it as painful, and requests discontinuation of the stimulus (Schiff et al. 2009).

#### VAS

The Visual Analog Scale (VAS) is a pain assessment tool where individuals rate their pain intensity by marking a point on a horizontal line anchored by "no pain" at one end (usually marked as 0) and "worst imaginable pain" at the other end (usually marked as 10). The individual marks a point on the line corresponding to their perceived level of pain intensity. The distance from the "no pain" end of the line to the marked point is then measured to quantify the pain intensity. The VAS is a commonly used method for assessing pain in clinical and research settings due to its simplicity and reliability. (Wong and Baker 1988).

#### WBFS

The Wong-Baker Faces Scale (WBFS) is a tool designed to help children communicate their experience of pain using facial expressions. It consists of a series of cartoon faces depicting different levels of pain intensity, ranging from a happy face indicating "no pain" to a very sad face indicating "worst pain." This scale allows children to point to the facial expression that best represents the level of pain they are experiencing. The scale has been widely used in pediatric settings and has been shown to be effective in helping children express their pain experiences. (Hicks et al. 2001).

### Desensitizing methods

The reviewed studies investigated various desensitizing methods for managing hypersensitivity in teeth affected by MIH. These methods include:

- Sealants: (Ballikaya et al. 2022), and (Bekes et al. 2022).
- Desensitizing pastes: (Bekes et al. 2017), (Ehlers et al. 2021), and (Pasini et al. 2018).
- Restorations: (Rolim et al. 2021).
- Full coverage crowns: (Singh et al. 2022).

Sealants utilized in the studies include resin-based sealants, glass ionomer, resin-modified glass ionomer, and polyacid-modified resin (Colombo and Beretta 2018). Desensitizing pastes and mousses contain ingredients, such as casein phosphopeptide-amorphous calcium phosphate (CPP-ACP), casein phosphopeptide-amorphous calcium fluoride phosphate (CPP-ACFP), hydroxyapatite, calcium glycerophosphate, self-assembling peptide, and fluoride (Enax et al. 2023). (Table 5).

### Hypersensitivity reduction

**Fig. 4:**
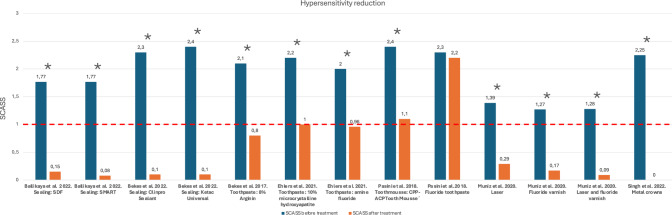
Bar chart of mean SCASS score before and after treatment among the included studies. SCASS sore ≥ 1 (dashed line) indicated clinically significant pain due to hypersensitivity. *Statistically significant difference

Most study groups reported an effective reduction in hypersensitivity, except when treating with fluoride varnish alone (Pasini et al. 2018). The most significant reduction was achieved with full coverage crowns (Singh et al. 2022). Sealants were also found to be effective in diminishing hypersensitivity, as evidenced by SCASS scale close to zero, indicating painless outcomes (Ballikaya et al. 2022; Bekes et al. 2022). Toothpastes and mousses marketed as desensitizing pastes yielded similar results across studies (Bekes et al. 2017; Ehlers et al. 2021; Pasini et al. 2018). (Fig. 4; Table 5).

### Follow-up time

The follow-up time varied substantially across the included studies, ranging from 8 weeks for desensitizing paste and to 2 years for full coverage crown therapy.

### *Meta*-analysis

**Fig. 5:**
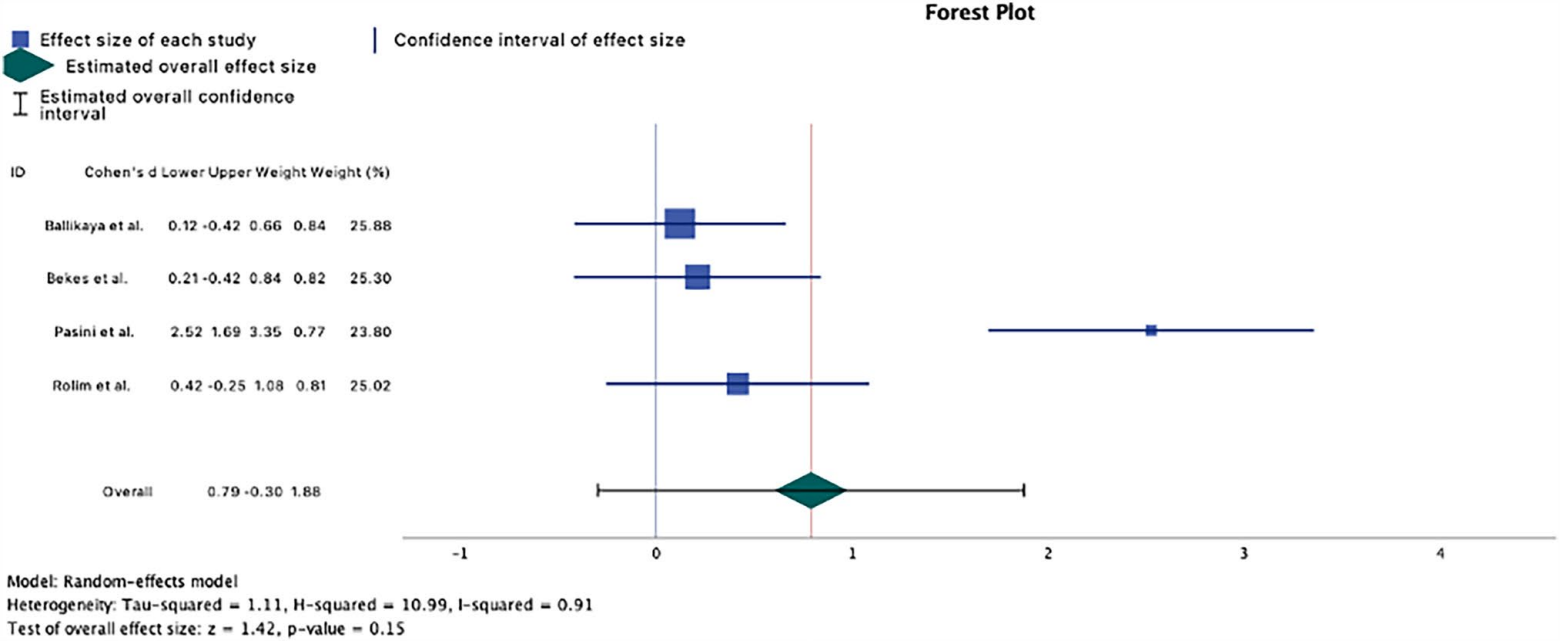
Forest plot of the mean difference of the hypersensitivity score between-study group and control group, together with the 95% confidence interval and the 95% prediction

With the exception of Pasini et al. (2018), all studies intersect zero, indicating that no statistically significant reduction in hypersensitivity can be observed between the control and intervention groups for the overall population (*p* > 0.05). The *I*^2^ value was 91%, indicating high between-study heterogeneity, which necessitated the use of a random-effects model. The test group demonstrated, on average, an additional 0.79 decrease in the SCASS score compared to the control group (95% CI − 0.30 to 1.68). However, due to the lack of significant heterogeneity (*p* > 0.05), it is not possible to determine the distinct effects of the different treatment modalities on the SCASS score and the reduction of hypersensitivity. Two of the articles (Bekes et al. 2017; Ehlers et al. 2021) did not provide a specified standard deviation, and Singh et al. (2022) lacked a control group, rendering them ineligible for inclusion in the meta-analysis. (Fig. 5).

## Discussion

Despite numerous comprehensive studies addressing various aspects of Molar-Incisor Hypomineralisation (MIH), there is a scarcity of literature describing effective management strategies for hypersensitivity in affected teeth. While many MIH studies highlight the suffering experienced by affected individuals, treatment methods are often lacking. The present review could only include eight studies, and the small sample sizes and limited follow-up times in these studies cast doubt on the reliability of advice regarding preferred treatments.

Furthermore, a few of the included studies (Ehlers et al. 2021) failed to report the degree of impairment of the treated hypersensitive MIH teeth, which is problematic as the severity of impairment affects the treatment needed. Teeth with enamel disintegration typically exhibit more symptoms and hypersensitivity (Jälevik and Möller 2007).

The management of MIH presents numerous challenges due to the broad spectrum of treatment modalities and the widely varying severity of affected teeth. These treatments range from preventive non-invasive approaches to invasive extractions, and, in some cases, include additional orthodontic treatment (Lygidakis et al. 2022). This review has focused on reducing hypersensitivity and examining which treatment modalities most effectively address the hypersensitivity of MIH-affected molars.

Silver diamine fluoride (SDF), as used in Ballikaya et al. (2022), is an effective agent for remineralization and stabilizing active carious lesions, which is particularly important as MIH molars are more frequently affected by caries (Weerheijm et al. 2001). SDF can be employed as a single chemotherapeutic non-invasive option or in combination with restorative treatments (Ballikaya et al. 2022). SDF reduces hypersensitivity by producing fluorohydroxyapatite, which blocks dentin tubules and increases mineral density (MacLean 2008). The SMART method used in Ballikaya et al. (2022) involves two steps: SDF treatment followed by sealing with high-viscosity glass ionomer cement, which enhances tissue remineralization and inhibits biofilm formation (Ballikaya et al. 2022).

Bekes et al. (2017) used casein phosphopeptide-amorphous calcium phosphate (CPP-ACP) to reduce hypersensitivity. Although there is no direct evidence supporting the treatment of MIH-affected molars with these products, it is empirically known that CPP-ACP interacts with fluoride ions, resulting in soluble fluoride, phosphate, and calcium ions that promote remineralization (Lygidakis 2010). Recent results suggest that CPP-ACP and 8% arginine can desensitize MIH-affected teeth. Arginine reduces hypersensitivity by interacting with calcium ions to create a calcium-rich layer that occludes the dentine tubules (Franca et al. 2015).

Calcium phosphate and hydroxyapatite particles act as reservoirs of calcium and phosphate, contributing to increased mineralization of the dentinal tubules (Ehlers et al. 2021). Fissure sealing is a preventive measure for controlling caries in MIH-affected teeth. Numerous clinical studies have documented the preventive effect on occlusal caries (Ahovuo-Saloranta et al. 2017). For children with MIH, simplifying the sealing procedures is crucial due to their increased risk of developing behavior management problems and dental fear (Jälevik & Klingberg 2002). Bekes et al. (2022) suggest that sealing can effectively reduce hypersensitivity in MIH-affected molars.

Conventional restorative treatment of MIH-affected teeth is indicated in the event of post-eruptive breakdowns or caries lesions (Lygidakis et al. 2022). Rolim et al. (2021) compared the success rates of restorative treatments using total-etch and self-etch composite systems and found significant reductions in hypersensitivity. However, there is no consensus in the current dental literature regarding the best restorative treatment for MIH-affected molars, indicating a need for further studies to determine long-term hypersensitivity reduction after restorative treatment.

Singh et al. (2022) compared the hypersensitivity before and after treatment of MIH-affected molars with post-eruptive breakdowns using three different crowns: Zirconia, lithium disilicate, and metal crowns. The success rates reported after 24 months were high, with no differences between the materials and a hypersensitivity rate of zero. A systematic review from 2022 promotes preformed metal crowns as an inexpensive option with high success rates and effective alleviation of hypersensitivity symptoms (Somani et al. 2022).

This review underscores the need for further research to identify the most effective long-term treatment strategies for reducing hypersensitivity in MIH-affected molars.

Upon comparing the included studies, it can be observed that there was an effective reduction of hypersensitivity in most study groups, except when treating with fluoride varnish alone (Pasini et al. 2018). The most significant reduction was achieved with full coverage crowns (Singh et al. 2022). Additionally, sealing proved to be an effective method in reducing hypersensitivity (Ballikaya et al. 2022; Bekes et al. 2022).

Desensitizing toothpastes and mousses yielded similar results across studies (Bekes et al. 2017; Ehlers et al. 2021; Pasini et al. 2018). However, drawing a unified conclusion from these studies is challenging due to variations in treatment schemes, follow-up times, and the diverse array of substances contained in the products used, such as silver diamine fluoride, casein phosphopeptide, amorphous calcium phosphate, hydrated silica, calcium carbonate, and 8% arginine, making it difficult to decipher the true effects of these treatments. A previous questionnaire study conducted among Swedish general dentists and pediatric dentists showed that a vast majority prefers to treat FPMs with mild defects using fluoride varnishes (Hajdarević et al. 2024).

Another challenge associated with the use of various toothpastes and mousses is that during follow-up assessments, some individuals exhibit SCASS values greater than 1, indicating painful responses to air-blast stimuli. Furthermore, comparing studies that employ different treatment alternatives is complicated by variations in the degree of MIH-affecting treated teeth. Research has demonstrated that children with teeth exhibiting post-eruptive disintegration experience more pain and hypersensitivity symptoms (Jälevik and Möller 2007). Therefore, it is advisable for studies investigating treatments for alleviating hypersensitivity to focus on MIH teeth with similar degrees of affection. This approach would help ensure more accurate comparisons and interpretations of treatment outcomes.

This is the first review exclusively addressing the treatment of hypersensitivity in MIH-affected teeth. A systematic review from 2021 investigated different treatment options for teeth in children affected by MIH (Somani et al. 2022). That review included four studies on the treatment of hypersensitivity in MIH-affected molars, all of which demonstrated a reduction in hypersensitivity post-treatment. However, none of the treatment modalities could be recommended due to moderate-to-high risk of bias, small sample sizes, and short follow-up times (Somani et al. 2022). The conclusions of this review align with those of Somani et al. (2022). The moderate risk of bias, small sample sizes, and short follow-up times in the included studies preclude drawing definitive conclusions regarding the most effective treatment modalities for reducing hypersensitivity in MIH-affected teeth.

## Conclusion

The present systematic review regarding the management of hypersensitivity in MIH-affected teeth has shown that there is a significant need for comparable qualitative studies to draw definitive conclusions.

The size of the study groups and the short period of follow-up in the existing studies limit the strength of the present conclusions showing that the treatment methods appearing more effective in reducing hypersensitivity are the sealing with resin composite or glass ionomer cement for MIH posterior mild cases, while crown therapy is more effective for severe cases.

## Data Availability

The data of this umbrella review will be shared on considerable request by the authors.
